# Deep Learning-Based Reconstruction vs. Iterative Reconstruction for Quality of Low-Dose Head-and-Neck CT Angiography with Different Tube-Voltage Protocols in Emergency-Department Patients

**DOI:** 10.3390/diagnostics12051287

**Published:** 2022-05-21

**Authors:** Marc Lenfant, Pierre-Olivier Comby, Kevin Guillen, Felix Galissot, Karim Haioun, Anthony Thay, Olivier Chevallier, Frédéric Ricolfi, Romaric Loffroy

**Affiliations:** 1 Department of Neuroradiology and Emergency Radiology, François-Mitterrand University Hospital, 14 Rue 7 Paul Gaffarel, BP 77908, 21079 Dijon, France; marc.lenfant@chu-dijon.fr (M.L.); pierre-olivier.comby@chu-dijon.fr (P.-O.C.); felix.gallissot@chu-dijon.fr (F.G.); frederic.ricolfi@chu-dijon.fr (F.R.); 2 Imaging and Artificial Vision (ImViA) Laboratory-EA 7535, University of Bourgogne/Franche-Comté, 9 10 Avenue Alain Savary, BP 47870, 21078 Dijon, France; kevin.guillen@chu-dijon.fr (K.G.); olivier.chevallier@chu-dijon.fr (O.C.); 3 Department of Vascular and Interventional Radiology, Image-Guided Therapy Center, François-Mitterrand 13 University Hospital, 14 Rue Paul Gaffarel, BP 77908, 21079 Dijon, France; 4 Computed Tomography Division, Canon Medical Systems France, 24 Quai Gallieni, 92150 Suresnes, France; karim1.haioun@medical.canon (K.H.); anthony.thay@eu.medical.canon (A.T.)

**Keywords:** deep-learning reconstruction, image quality, CT angiography, low-dose CT scan, emergency

## Abstract

Objective: To compare the image quality of computed tomography angiography of the supra-aortic arteries (CTSA) at different tube voltages in low doses settings with deep learning-based image reconstruction (DLR) vs. hybrid iterative reconstruction (H-IR). Methods: We retrospectively reviewed 102 patients who underwent CTSA systematically reconstructed with both DLR and H-IR. We assessed the image quality both quantitatively and qualitatively at 11 arterial segmental levels and 3 regional levels. Radiation-dose parameters were recorded and the effective dose was calculated. Eighty-six patients were eligible for analysis Of these patients, 27 were imaged with 120 kVp, 30 with 100 kVp, and 29 with 80 kVp. Results: The effective dose in 120 kVp, 100 kVp and 80 kVp was 1.5 ± 0.4 mSv, 1.1 ± 0.3 mSv and 0.68 ± 0.1 mSv, respectively (*p* < 0.01). Comparing 80 kVp + DLR vs. 120 and 100 kVp + H-IR CT scans, the mean overall arterial attenuation was about 64% and 34% higher (625.9 ± 118.5 HU vs. 382.3 ± 98.6 HU and 468 ± 118.5 HU; *p* < 0.01) without a significant difference in terms of image noise (17.7 ± 4.9 HU vs. 17.5 ± 5.2; *p* = 0.7 and 18.1 ± 5.4; *p* = 0.3) and signal-to-ratio increased by 59% and 33%, respectively (37.9 ± 12.3 vs. 23.8 ± 9.7 and 28.4 ± 12.5). This protocol also provided superior image quality in terms of qualitative parameters, compared to standard-kVp protocols with H-IR. Highest subjective image-quality grades for vascular segments close to the aorta were obtained with the 100 kVp + DLR protocol. Conclusions: DLR significantly reduced image noise and improved the overall image quality of CTSA with both low and standard tube voltages and at all vascular segments. CT that was acquired with 80 kVp and reconstructed with DLR yielded better overall image quality compared to higher kVp values with H-IR, while reducing the radiation dose by half, but it has limitations for arteries that are close to the aortic arch.

## 1. Introduction

Computed tomography angiography (CTA) is the first-line imaging study for a growing number of neurovascular diseases. Its unrivalled availability, minimal invasiveness, high resolution, and combination if needed with cerebral perfusion CT make it a versatile imaging tool for rapid vascular assessment. The main disadvantage of CT is the delivery of ionizing radiation to the patient. The increasing use of CT has resulted in greater cumulative doses [1,2,3,4]. The As Low As Reasonably Achievable (ALARA) principle is applied to minimize radiation exposure during scheduled CTs; however, regarding emergency CTs, studies are needed to assess the efficacy and reliability of low-dose protocols [5,6,7,8].

Emergency CTA can have a major impact on treatment decisions in patients with acute neurologic symptoms. Therefore, high image quality and fast reconstruction are needed in this situation. Moreover, a challenge raised by CTA of the supra-aortic arteries is that complete imaging from the aortic arch to the circle of Willis is performed in a single acquisition, although the arteries are in three anatomically complex regions that are characterized by different attenuation profiles, namely, the shoulders, neck, and skull. Thus, physics-based artifacts may considerably impair image quality, thereby reducing diagnostic confidence [9].

Historically, diverse approaches have been suggested to improve or maintain the image quality of head-and-neck CT scans while minimizing the radiation dose. Examples include the use of lower or adjusted tube voltages, tube-current modulation, and the application of iterative reconstruction (IR) algorithms [10]. Tube-voltage reduction is of special interest as the radiation dose varies inversely with the third power of kVp [11] and iodine attenuation increases when photon energy approaches the K-edge of iodine (33.2 keV) [12,13]. On the other hand, lowering photon energy inevitably increases noise and beam hardening artifacts, requiring efficient reconstruction algorithms to maintain diagnostic image quality. Several studies have already compared low-kVp CT of the supra-aortic arteries with IR vs. standard-kVp CT with filtered back-projection (FBP) [14,15,16,17]. Image quality was generally better with low-dose CT and IR, although limitations for evaluating arterial segments close to the aortic arch or intracranial arteries were observed in some studies [7,15]. These discrepancies may be ascribable to differences in IR algorithms, some of which incorporate image-based anatomical models [18], and in the number and variety of vascular segments assessed.

Most recently, artificial intelligence methods were introduced into the field of CT reconstruction. Two deep learning-based reconstruction techniques (DLRs) have been approved by the Food and Drug Administration and European Union, Advanced Intelligent Clear-IQ Engine (AiCE) (Canon Medical, Otawara, Japan) and TrueFidelity (GE Healthcare, IL, USA) [19,20]. Both use a deep convolutional neural network to distinguish true signal from noise within the images. The deep convolutional neural network used in AiCE was trained to restore image quality equivalent to that produced by advanced model-based iterative reconstruction (MBIR) from paired low-dose CT images that are processed using hybrid iterative-reconstruction (H-IR). Thus, AiCE was designed to provide the noise reduction and increased spatial resolution available with MBIR but within a far shorter time, consistent with the needs of emergency patients.

Data on the properties and clinical usefulness of DLR in neuroradiology are scarce [21,22]. Given that MBIR outperformed H-IR for low-kVp CT in numerous studies [23,24,25,26] we hypothesized that DLR might improve neurovascular imaging quality in emergency settings.

Here, our objective was to compare DLR and H-IR regarding the quality of supra-aortic-artery CT imaging with various tube-voltage protocols in low dose settings used as part of our neurovascular imaging protocol in an emergency department.

## 2. Materials and Methods

### 2.1. Study Population

We conducted a retrospective single-center study of consecutive patients who were referred to our emergency department between January 2021 and February 2021 and who underwent CT of the supra-aortic arteries. This period was chosen as we updated our standard CTA protocol on the 1 February by extending the automatic tube-voltage selection ^SURE^kV range from 100–120 kVp to 80–120 kVp. At that time, our “stroke-protocol” was standardized and included a fixed 120 kVp CTA. These CTA have also been included in this study. We identified the patients using our radiology information system’s built-in thesaurus (XPLORE; EDL, La Seyne-Sur-Mer, France). For each search, keywords related to supra-aortic arteries (“carotid” OR “vertebral”) and to predefined kVp settings (80 kVp OR 100 kVp OR 120 kVp) were combined with CTA as a filter using the Boolean operator “AND”. Exclusion criteria were, allergy to iodinated contrast, severe renal disease, age < 18 years, nonstandard supra-aortic-artery CT protocol, body mass index (BMI) above 35, and health condition responsible for bilaterally altered hemodynamics. All data were anonymized. In compliance with French law about retrospective studies of anonymized data, neither ethics committee approval nor patient informed consent were required.

### 2.2. CT Scanning Protocol

All CT examinations were performed with a 320-detector row CT scanner (Aquilion ONE GENESIS, Canon Medical). For each CT, the main parameters were as follows: helical acquisition, 0.5 mm × 80 rows; beam pitch, 0.813; gantry rotation time, 0.35 s; matrix, 512 × 512; and field of view, 320 mm. Tube voltage was 120 kVp, 100 kVp, or 80 kVp. Automatic tube-current modulation (SUREExposure™ 3D, Canon Medical) was used for all CTs. The noise-index settings were based on our initial experience in clinical practice and on the manufacturer’s recommendations. Reconstruction parameters were 1.0-mm slice thickness and 0.8-mm gap. Table 1 lists the acquisition and reconstruction parameters. In accordance with our standard CTA protocol involving subtraction, all patients received a fixed 65-mL intravenous bolus of Iomeron, 350 mg iodine per mL (Bracco Imaging, Courcouronnes, France), followed by a 50-mL bolus of saline at an injection rate of 4 mL/s for both. Image acquisition was triggered using a predefined threshold of aortic-arch attenuation. Each CT scan was reconstructed using both H-IR (AIDR 3D) with a standard FC43 kernel and DLR with beam-hardening correction (AiCE Body Sharp).

### 2.3. Image-Quality Assessment

#### 2.3.1. Objective Quantitative Image-Quality Assessment

The images were analyzed on a dedicated workstation (Vitrea, version 6.5.3; Vital, Minneapolis, MN, USA) by an independent radiologist who had 5 years of experience in neuroradiology and was blinded to the reconstruction method and kVp value. The reader manually placed circular regions of interest (ROI) within the axial images.

ROIs were as large as possible while avoiding regions with severe artifacts (e.g., caused by dental artifacts or motion, flow artifacts, or partial volume effects). ROIs were then duplicated on the two image sets. Mean attenuation with the standard deviation (Hounsfield Units [HU]) was obtained for each measurement. Measurements were performed at the aortic arch (AO), common carotid artery (CCA), cervical internal carotid artery (ICAc), intrapetrous ICA (ICAp), terminal ICA (ICAt), middle cerebral artery (MCA), vertebral artery (portions V1, V2, V3, and V4), and basilar artery (BA). Vascular regions were also categorized as close to the aortic arch (AO, CCA, and V1), close to bones (ICAp, V2, and V3), or intracranial (ICAt, MCA, V4, and BA).

The signal-to-noise ratio (*SNR*) and contrast-to-noise ratio (*CNR*) were calculated based on the following equation [15]:

$$SNR=\frac{S_{vessel}}{N_{vessel}}$$

$$CNR_{ec}=\frac{S_{vessel}-S_{muscle}}{\left(N_{vessel}+N_{muscle}\right)/2}\;\;\;\;CNR_{ic}=\frac{S_{vessel}-S_{brain}}{\left(N_{vessel}+N_{brain}\right)/2}$$

where *S_vessel_* was the mean signal intensity of the vessels of interest. When an intracranial abnormality was responsible for hemodynamic alterations, the ROI was placed in a contralateral vessel segment. *S_muscle_* was calculated as the mean attenuation of neck muscles on both sides in areas as free of fat as possible. *S_brain_* was the mean attenuation in an ROI of about 1 cm^2^ positioned within the white matter of the centrum semiovale. Noise was defined as the mean of the standard deviations of these measurements.

*CNR_ec_* and *CNR_ic_* were the CNRs in extracranial and intracranial vessels, respectively.

#### 2.3.2. Subjective Qualitative Image-Quality Assessment

All supra-aortic-artery CTs were randomized, anonymized, and independently evaluated on standard LCD monitors by two radiologists with 4 and 10 years of experience, respectively, who were blinded to all patient data and reconstruction parameters. Both readers used 1–5 Likert scales to subjectively rate perceived diagnostic confidence, vessel sharpness, artifacts, and noise level on axial slices within predefined regions and overall (Table 2).

During the evaluation, readers were free to adjust the window level and width. To improve interobserver agreement, the readers were trained blindly on images obtained from patients excluded from the study.

### 2.4. Radiation Dose Measurements

To assess radiation exposure we recorded the volume CT dose index (CTDIvol) and dose-length product (DLP) recorded as Digital Imaging and Communications in Medicine (DICOM) data. Equivalent doses (EDs) were calculated after scanning by multiplying the DLP by the k factor (0.00751 for the CTSA) [27].

### 2.5. Statistical Analysis

Continuous variables were described as mean ± standard deviation (SD) and dichotomous variables as number (percentage). The Shapiro-Wilk test showed that all parameters were normally distributed. Therefore, we applied the two-sided paired *t*-test to compare H-IR (AIDR 3D) and DLR (AiCE) images within each group regarding the mean attenuation value (HU), noise (SD), SNR, CNR, and image-quality scores. The homoscedastic *t*-test was used for intergroup comparisons. *p* values smaller than 0.05 were considered statistically significant. Interobserver agreement for qualitative image-quality scores was evaluated using weighted Cohens’ kappa coefficients (k), interpreted as follows: <0, no agreement; 0.00–0.20, poor agreement; 0.21–0.40, fair agreement; 0.41–0.60, moderate agreement; 0.61–0.80, substantial agreement; and 0.81–1.00, excellent agreement. All statistical tests were performed using STATA^®^ software version 13 (StataCorp, College Station, TX, USA) and Excel version 2016 (Microsoft, Redmond, WA, USA).

## 3. Results

### 3.1. Patient Characteristics

**Figure 1:**
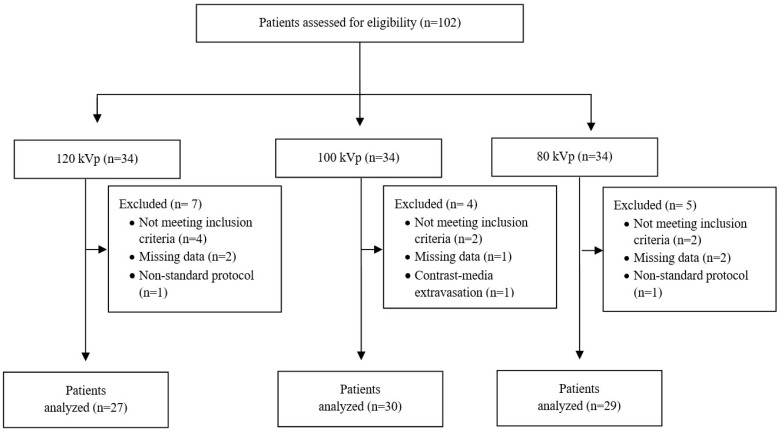
Flow chart of the study. kVp, kilovoltage peak.

Of 102 consecutive eligible patients, 86 (33 men and 53 women) were included in the analysis (Figure 1). The mean age was 64.8 ± 18.3 years (range, 24–92 years) and the mean BMI was 25.4 ± 4 kg/m^2^ (range, 16.5–34.4 kg/m^2^). Age, BMI, and sex distribution did not differ significantly between the three groups.

### 3.2. Radiation Exposure

The mean CTDIvol and DLP values were lowest with 80 kVp and highest with 120 kVp (Table 3). The ED was significantly lower with 80 kVp than with 100 or 120 kVp (0.68 ± 0.1 mSv vs. 1.1 ± 0.3 mSv 1.5 ± 0.4 mSv, respectively, *p* < 0.01).

### 3.3. Quantitative Image Analysis

We analyzed 9272 measurements (means and SDs for 4636 ROIs). The quantitative image analysis results at segmental levels are summarized in the Supplementary Materials.

#### 3.3.1. Image Signal Fluctuation with DLR vs. H-IR

**Figure 2:**
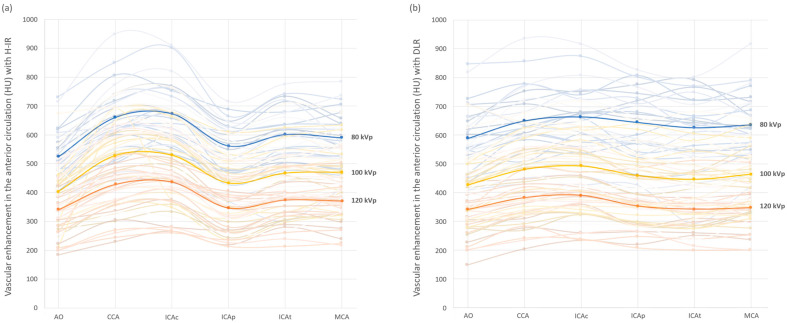
Arterial vascular attenuation profile for the anterior cerebral vascularization with H-IR (**a**) and DLR (**b**), with 80 kVp in blue, 100 kVp in yellow and 120 kVp in orange. Mean values are displayed in bold lines. Vessel contrast for each patient are in the corresponding light colors. AO: Aortic arch; CCA: common carotid artery; ICAc: cervical internal carotid artery; ICAp: intrapetrous internal carotid artery; ICAt: terminal internal carotid artery; MCA: middle cerebral artery.

Figure 2 shows the mean arterial vascular attenuation for the anterior circulation at different kVp with both DLR and H-IR. These data showed fluctuations in terms of image signal between the two reconstruction methods depending on the ROI location and the kVp selected. In fact, overall vascular enhancement with DLR compared to H-IR was lower by 6% with 120 kVp and higher by 5% with 80 kVp. These differences were maximal in low-kVp settings and reached +65 HU (+12%) at the aorta and +82 HU (+15%) at the intrapetrous portion of the carotidal artery with DLR vs. H-IR.

#### 3.3.2. Comparison of the Two Reconstruction Techniques at Constant kVp Value

As shown in Table 4, noise was significantly lower on the DLR images at each ROI location and kVp value. Relative noise reduction was greatest in areas close to the AO and to bones (−36% and −30%, respectively) and minimal for the intracranial arteries (−22%). The relative overall noise reduction with DLR was constant across all three kVp values.

SNR and CNR were significantly higher on DLR images at each ROI location. At regional levels, SNR gains with DLR were greatest in regions close to the AO and to bones (+43% and +42%, respectively) and were smaller for the intracranial arteries (+28%).

#### 3.3.3. Comparison of Low kVp plus DLR vs. Standard kVp plus DLR

When using DLR, the image signal and noise were significantly greater with 80 kVp than with 100 or 120 kVp (Table 5). Decreasing the tube voltage from 120 kVp or 100 kVp to 80 kVp increased the image signal by +75% and +37%, and the image noise by +40%. This resulted in significantly higher SNR and CNR values at the 80 kVp vs. 120 kVp. There were no significant differences at segmental and regional levels in term of SNR or CNR between 80 kVp and 100 kVp CT-scans with DLR as the image noise increase counterbalanced the iodine attenuation increase.

#### 3.3.4. Comparison of Low kVp plus DLR vs. Standard kVp plus H-IR

We compared 80-kVp images that were taken with the DLR to 100- and 120-kVp images with H-IR (Table 6). Noise was significantly greater in the 80-kVp plus DLR group for the ICAt, MCA, and BA and was lower for the aorta, and the V1 segment of the vertebral artery.

At regional levels, noise was significantly less in the vascular segments close to the AO in the 80 kVp plus DLR group, compared to the 100 kVp and 120 kVp plus H-IR groups (−17% and −20%, respectively). On the other hand, with 80 kVp plus DLR, noise at the intracranial arteries was significantly greater than with 100 kVp or 120 kVp plus H-IR (+14% and +13%, respectively).

SNR and CNR were consistently higher at regional levels with 80 kVp plus DLR than with 100 and 120 kVp plus H-IR but they did not reach significant levels for the segmental analysis vs. 100 kVp plus H-IR at the CCA, ICAc, ICAt, or MCA.

Considering the overall vascular enhancement, no significant difference was found between the three groups while the image signal reached 625.9 ± 118.5 HU with 80 kVp plus DLR, vs. 468 ± 118.5 and 382.3 ± 98.6 HU with 100 kVp and 120 kVp plus H-IR, respectively. This led to a significant increase in terms of both SNR and CNR for the former group reaching, respectively, 37.9 ± 12.3 and 39.6 ± 10.4, vs. 28.4 ± 12.5, and 26.9 ± 9.5 with 100 kVp + H-IR, and 23.8 ± 9.7 and 22.1 ± 7.5 with 120 kVp + H-IR.

### 3.4. Qualitative Image Analysis

As shown in Table 7, DLR yielded the highest image-quality scores across kVp values and vascular regions. The inter-observer agreement was moderate (κ, 0.42; weighted κ, 0.53).

**Figure 3:**
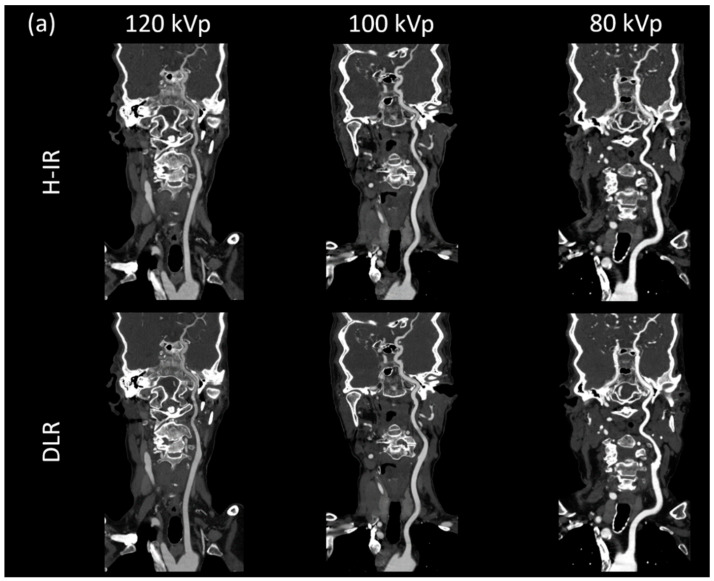

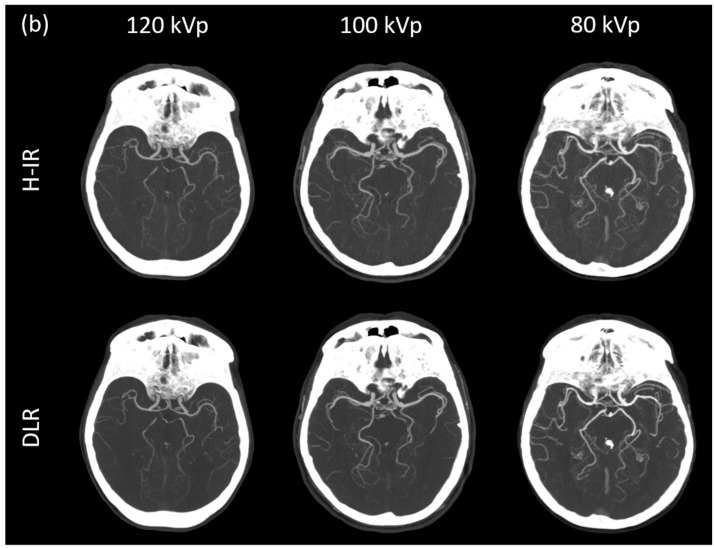
(**a**) Curved planar reconstructed CT image, (**b**) Axial MIP 20mm of patients from each group for both DLR (AiCE) and H-IR (AIDR 3D) reconstructions with a constant window setting (W:1000 L:300). 120 kVp patient was a 68-year-old woman (BMI: 25.7), CTDI 4.7 mGy, DLP 196.8 mGy·cm.; 100 kVp patient was an 86-year-old woman (BMI: 25.2), CTDI 4.7 mGy, DLP 176.6 mGy·cm; 80 kVp patient was a 79-year-old woman (BMI: 23.8), CTDI 2.5 mGy, DLP 88.3 mGy·cm. 80 kVp image provided increased contrast with both DLR and H-IR. Note that DLR maintain a noticeably low noise level for the 3 groups.

Confidence in diagnosis, perceived sharpness and noise reduction were significantly higher with DLR in the entire cohort, whatever the tube-voltage and ROI location (Figure 3).

**Figure 4:**
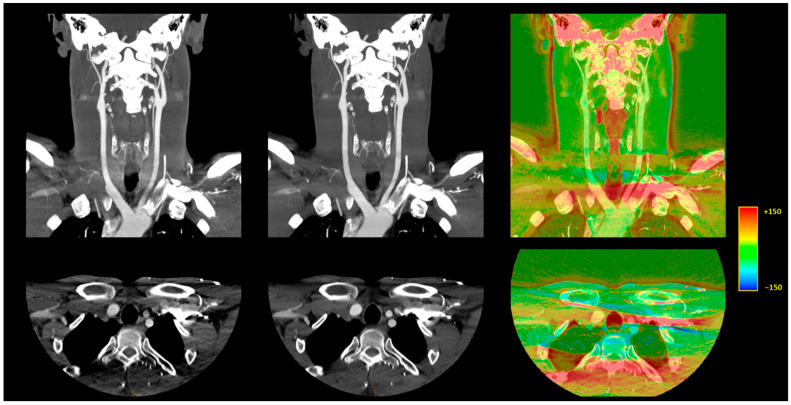
Illustration of the differences between DLR and H-IR for the vascular regions close to the aortic arch. A 10 mm, maximum intensity projection, coronal CT reconstruction (**top row**) and 1-mm axial CT reconstruction (**bottom row**). **Left**: AIDR 3D, hybrid iterative reconstruction. **Middle**: AiCE, deep learning-based reconstruction. **Right**: Color-mapped subtraction [AiCE-AIDR 3D] layered on the DLR image to visually highlight the differences between the two reconstruction techniques.

Subjective noise reduction was greatest with 80 kVp and for vessels close to the aorta (4.4 ± 0.6 with DLR vs. 3.6 ± 0.8 with H-IR). Increases in term of diagnostic confidence were also the highest for vessels close to the aortic arch with 80 kVp CTA + DLR (4.4 ± 0.6 vs. 3.6 ± 0.8 with H-IR). Artifacts were maximal in this setting with a score of 2.9 ± 1.1 with DLR and 2.5 ± 1.2 with H-IR (Figure 4). Overall, artifact reduction was slightly better with DLR, although the difference did not reach significant levels for the intradural arteries.

Comparing 80 kVp plus DLR to 100 and 120 kVp plus H-IR protocols, confidence significantly increased (*p* < 0.05) in all segments that were evaluated, except for segments too close to the aorta.

Figure 4 depicts the differences between DLR and H-IR for the vascular regions close to the aortic arch.

## 4. Discussion

This study provides the first evidence on the effects of DLR on image quality and radiation dose when performing CTA of the supra-aortic arteries in the emergency setting. We retrospectively evaluated image quality for 11 vascular segments and three anatomical regions in 86 patients imaged using three different tube voltages (120, 100, and 80 kVp) at low doses. The mean CTDIvol at 80-kVp CT in the current study was only 2.3 ± 0.2 mGy which is lower than previously reported values.

DLR yielded significantly less noise and higher SNR and CNR values than H-IR with all three voltages and at all ROI locations. Image quality that was assessed qualitatively was also significantly better with DLR than with H-IR.

The AiCE DLR applies the advanced capabilities of a trained deep convolutional neural network to suppress noise, thus generating high-quality images. With all three tube voltages, the overall vascular image noise was lower by 29% with DLR, and the SNR and CNR values were higher by 36% and 42%, respectively. The noise reduction capabilities of AiCE varied across ROI locations, from 36% in vascular segments close to the AO to 30% in segments close to bone and 22% in intracranial arteries. We also found slight but significant image signal variations with DLR vs. H-IR, depending on the tube voltage and ROI location. Although we did not have ground truth HU values in vivo, DLR seemed more robust in maintaining the signal information, resulting in greater consistency with adjacent vascular segments, notably at low kVp settings. In fact, in low-kVp settings, these variations reached +65 HU (+12%) at the AO and +82 HU (+15%) at the ICAp with DLR vs. H-IR, suggesting a reduced attenuation loss secondary to beam-hardening in these segments with DLR. We believe these variations were not produced by the neural network—which is a denoising algorithm [28]—but rather by the entire AiCE in which both the raw-data and the image-domain components might contribute to the measured HU value variation.

Tube-voltage reduction also drastically increased the iodine attenuation. This well-known phenomenon is due to decreased Compton scattering and a stronger photoelectric effect when the photon energy approaches the K-edge of iodine (33.2 keV) [11,13]. In our study, the increase in image signal and reduction in radiation dose obtained by decreasing the tube voltage from 120 or 100 kVp to 80 kVp were consistent with earlier data [14,15].

**Figure 5:**
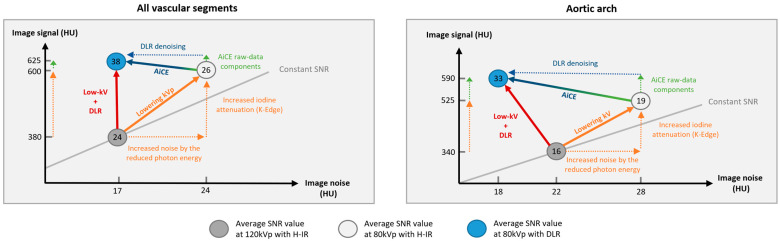
Schematic representation of the low-tube voltage approach combined with deep learning reconstruction to increase the signal-to-noise ratio vs. standard-kVp and iterative reconstruction protocol.

We then compared 80 kVp plus DLR to 120 and 100 kVp plus H-IR. Although there was no significant difference in image noise for overall vascular attenuation, several noteworthy differences were found at the segmental and regional levels (Figure 5). First, the image noise was lower with 80 kVp plus DLR in regions usually described as difficult to image, i.e., vessels close to the AO or to bone [7,15]. Second, the mean vascular noise in the intracranial arteries was higher with 80 kVp plus DLR than with 100 or 120 kVp plus H-IR (20.6 ± 5.5 HU vs. 18.0 ± 5.5 HU [+14%] and 18.3 ± 5 HU [+13%] at 120 kVp and 100 kVp, respectively). Although this noise increase of less than 3 HU is of negligible clinical relevance, especially since the SNR remained 21% higher, it may suggest that the maximum denoising capabilities of DLR were reached for intracranial vessels at 80 kVp. Third, despite the superior image quality in terms of qualitative parameters with DLR at lower-kVp we found that vascular segments close to the aorta remained the most subject to artifacts at 80 kVp with the lowest image quality score of 2.9 ± 0.6 with DLR vs. 3.4 ± 0.6 and 3.5 ± 0.6 with H-IR, 3.6 ± 0.5, and 3.8 ± 0.6 with DLR at 100 and 120 kVp, respectively. These streak artefacts were only slightly diminished by AiCE and were caused by the increased arterial enhancement at 80 kVp of the non-utilized contrast agent within the brachiocephalic vein.

Our findings confirm the superiority of DLR over conventional H-IR for CTA of all supra-aortic artery segments using three tube voltages. Our results are consistent with studies of pulmonary and abdominal CT scans that were reconstructed using DLR algorithms [29,30,31,32]. We also demonstrated that low-kVp CT with DLR provided better image quality as assessed quantitatively (SNR and CNR) and qualitatively, compared to higher kVp values with H-IR in most vessels. Last, our data indicate that although 80-kVp protocols with DLR proved feasible and reliable, optimal image quality of the intracranial arteries and arteries that are close to the aortic arch may be obtained with 100 kVp + DLR as the qualitative measurements were highest in this setting.

The limitations of our study should be addressed. First, we recruited all patients at a single center. Second, with a mean BMI of 25.3 ± 4.0 kg, our cohort was representative of the normal weight range. Additional studies are warranted to evaluate whether our results apply to obese patients in whom photon-starvation might impair image quality. Further, tube-current modulation (SUREExposure) frequently reached its lower limit at 120 kVp. Given our low-dose settings, this led to the high-kVp CTs being performed with an inappropriately low tube current. The result was an overall decrease in image quality at 120 kVp with both reconstruction methods. This suggests that a low-dose CT scan should preferably be achieved by reducing the tube-voltage and maintaining an adequate tube current, rather than performing a high-kVp and extremely low mAs. Third, most of the 120 kVp CTA were acquired as part of our institutional “stroke-protocol” which may have further degraded the image quality by including patients with more severe conditions. This bias has been partially addressed by placing ROIs in contralateral vessels to the ones with potentially altered hemodynamics. Fourth, we did not assess the potential superiority of DLR over H-IR for detecting neurovascular disease due to the considerable heterogeneity of the suspected diagnoses in both groups. Instead, the goal of our study was to evaluate the impact on the image quality of DLR with different tube voltages. Fifth, mean vascular enhancement was 625.9 ± 118.5 HU in the 80-kVp group which was far above the diagnostic limit of 250 HU. This implies that a decrease in the contrast agent dose should be achievable with 80 kVp and DLR, a possibility of interest for patients with impaired renal function which may also improve the image quality by reducing the streak artifacts induced by a high contrast media concentration in the subclavian vein. Studies are under way to further assess this hypothesis. Sixth, although blinded to all reconstruction parameters, reviewers could have easily differentiated the two algorithms as the image texture noticeably changed, introducing a potential bias. Last, our study compared two reconstruction algorithms (AIDR 3D and AiCE) that were developed by the same manufacturer (Canon Medical). Comparisons of IR algorithms from different manufacturers to DLR may be warranted. Nonetheless, the comparability of the three tube-voltage groups and the evaluation of both objective (SNR, CNR, CTDI, and DLP) and subjective parameters support the validity of our findings.

## 5. Conclusions

DLR significantly reduced image noise and improved the image quality of CTSA performed in the emergency setting, vs. H-IR. Thus, low-dose CTSA with 80 kVp + DLR yielded better overall image quality than higher-kVp CT with H-IR while halving the radiation dose, but it had limitations for imaging arteries close to the aortic arch.

## Figures and Tables

**Table 1:** CT acquisition parameters in each peak kilovoltage (kVp) group.

Parameters	120 kVp	100 kVp	80 kVp
Acquisition mode	Helical	Helical	Helical
Tube voltage (kVp)	120	100	80
Tube current range (mA)	200–700	200–700	200–700
Collimation (mm)	0.5 × 80	0.5 × 80	0.5 × 80
Rotation time (s)	0.35	0.35	0.35
Field of view (mm)	320	320	320
Slice thickness (mm)	1	1	1
Interval (mm)	0.8	0.8	0.8
Pitch	0.8	0.8	0.8
Noise index	10	10	10

mA, milliampere; mm, millimeter; s, second.

**Table 2:** Five-point Likert-scale used for qualitative image evaluation.

Grading	Confidence	Artifacts	Sharpness	Noise
1	Non-diagnostic	Extensive	Very blurry	Very high/Very coarse graininess
2	Low confidence	Significant	Blurry	High/Coarse graininess
3	Average	Average	Average	Average
4	Good confidence	Few	Sharp	Low/Fine graininess
5	Highest confidence	Very few	Very Sharp	Very low/Very fine graininess

**Table 3:** Comparison of radiation dose to the patient with the three kVp values.

Parameters	120 kVp (*N* = *27*)	100 kVp (*N* = *30*)	80 kVp (*N* = *29*)	100 kVp vs. 120 kVp		80 kVp vs. 100 kVp		80 kVp vs. 120 kVp	
				% Change	*p*-Value	% Change	*p*-Value	% Change	*p*-Value
Age	67 ± 17.7	65.7 ± 19	63.4 ± 18.8	*NS*	0.96	*NS*	0.64	*NS*	0.67
Female/male	14/13	18/12	21/8	*NS*	0.54	*NS*	0.32	*NS*	0.12
BMI, kg/m^2^	25.6 ± 4.6	25 ± 4.1	25.6 ± 3.3	*NS*	0.55	*NS*	0.48	*NS*	0.99
Right/left arm injection	14/13	14/16	14/15	*NS*	0.7	*NS*	0.9	*NS*	0.8
CTDI_vol_, mGy	4.8 ± 1.1	4 ± 1.1	2.3 ± 0.2	−17%	0.01	−42%	<0.01	−51%	<0.01
DLP, mGy.cm	197.1 ± 51.2	150.7 ± 37.8	90.4 ± 9.4	−24%	<0.01	−40%	<0.01	−54%	<0.01
ED, mSv	1.5 ± 0.4	1.1 ± 0.3	0.68 ± 0.1	−24%	<0.01	−40%	<0.01	−54%	<0.01

**Table 4:** Comparison of the two reconstruction techniques at regional levels with constant kVp value.

Parameters, *N*	120 kVp, 27			100 kVp, 30			80 kVp, 29			All kVp, 86		
	H-IR	DLR	*p*-Value	H-IR	DLR	*p*-Value	H-IR	DLR	*p*-Value	H-IR	DLR	*p*-Value
Close to the aorta												
Image signal (HU)	382.8 ± 115.8	351.1 ± 102.4	<0.01	474.3 ± 127.7	453.6 ± 115.4	<0.01	580.9 ± 124	595.8 ± 122.7	<0.01	475.3 ± 144.3	465.8 ± 150.4	<0.01
Image noise (HU)	18.9 ± 4.7	12.2 ± 1.9	<0.01	19.4 ± 6.1	12 ± 2.7	<0.01	23.5 ± 6.1	15.6 ± 3.1	<0.01	20.8 ± 5.9	13.4 ± 3.1	<0.01
SNR	21.9 ± 9.9	29.5 ± 10.2	<0.01	28.1 ± 15.8	39.9 ± 15.3	<0.01	27 ± 11.5	39.7 ± 12.4	<0.01	25.1 ± 12.5	36 ± 13.3	<0.01
CNR	20.5 ± 8.9	27.5 ± 10.6	<0.01	25.3 ± 8.0	38.3 ± 14	<0.01	26.5 ± 10.1	39.5 ± 11.2	<0.01	24 ± 10.4	34.9 ± 12.9	<0.01
Close to bones												
Image signal (HU)	384.2 ± 95.2	371.3 ± 86.4	<0.01	490.2 ± 117.4	486 ± 117.1	0.2	612.1 ± 114	647.8 ± 121.4	<0.01	491.3 ± 142.2	501.1 ± 157.2	<0.01
Image noise (HU)	16.9 ± 4.8	11.7 ± 2.7	<0.01	17 ± 4.6	11.9 ± 3.3	<0.01	22.7 ± 5.5	15.8 ± 3.2	<0.01	19.1 ± 5.7	13.3 ± 3.6	<0.01
SNR	24.2 ± 8.4	33.4 ± 11.3	<0.01	30.7 ± 11.2	43.1 ± 15.3	<0.01	28 ± 6.8	42.7 ± 12.6	<0.01	27.2 ± 6.8	39.2 ± 13.7	<0.01
CNR	22 ± 7.8	30.9 ± 11.4	<0.01	27.6 ± 6.3	40.6 ± 11.7	<0.01	27.8 ± 6.2	44.8 ± 11.7	<0.01	40.1 ± 6.2	57.2 ± 29.4	<0.01
Intra-dural arteries												
Image signal (HU)	366.7 ± 79.3	345.1 ± 79.1	<0.01	456.4 ± 106.9	450.1 ± 107.4	<0.01	584.7 ± 107.3	618.7 ± 116	<0.01	470.9 ± 133.2	472.9 ± 152.6	0.3
Image noise (HU)	18 ± 5.5	14.2 ± 4.7	<0.01	18.3 ± 5	13.9 ± 3.5	<0.01	26.3 ± 7.3	20.6 ± 5.5	<0.01	20.9 ± 7.2	16.3 ± 5.6	<0.01
SNR	22.1 ± 8.5	26.2 ± 8.9	<0.01	26.5 ± 8.8	34.5 ± 12.7	<0.01	23.8 ± 7.3	32 ± 10.1	<0.01	24.1 ± 8.3	30.9 ± 11.2	<0.01
CNR	21.7 ± 6.8	27.1 ± 10.3	<0.01	26.0 ± 6.0	35.9 ± 9.0	<0.01	24.4 ± 5.6	36.0 ± 9.2	<0.01	29.1 ± 11.6	38.8 ± 13.6	<0.01
All vascular segments												
Image signal (HU)	382.3 ± 98.6	358 ± 90.2	<0.01	468 ± 118.5	456.6 ± 113.5	<0.01	597.6 ± 114.9	625.9 ± 118.5	<0.01	484.9 ± 141.7	482.8 ± 154.4	0.16
Image noise (HU)	17.5 ± 5.2	12.6 ± 3.6	<0.01	18.1 ± 5.4	12.6 ± 3.2	<0.01	24.4 ± 6.7	17.7 ± 4.9	<0.01	20 ± 6.6	14.3 ± 4.6	<0.01
SNR	23.8 ± 9.7	30.3 ± 11.1	<0.01	28.4 ± 12.5	38.7 ± 14.7	<0.01	26.4 ± 9.1	37.9 ± 12.3	<0.01	26.3 ± 10.7	35.8 ± 13.4	<0.01
CNR	22.1 ± 7.5	28.9 ± 9.5	<0.01	26.9 ± 9.5	38.1 ± 13.3	<0.01	26.4 ± 7.7	39.6 ± 10.4	<0.01	25.2 ± 8.6	35.7 ± 12.2	<0.01

**Table 5:** Quantitative image analysis at regional levels of low-kVp protocol with DLR vs. higher-kVp protocol with DLR.

Parameters	120 kVp + DLR	100 kVp + DLR	80 kVp + DLR	80 kVp + DLR vs.			
				120 kVp + DLR		100 kVp + DLR	
				% Change	*p*-Value	% Change	*p*-Value
Close to the aorta							
Image signal (HU)	351.1 ± 102.4	453.6 ± 115.4	595.8 ± 122.7	+70%	<0.01	+31%	<0.01
Image noise (HU)	12.2 ± 1.9	12 ± 2.7	15.6 ± 3.1	+28%	<0.01	+30%	<0.01
SNR	29.5 ± 10.2	39.9 ± 15.3	39.7 ± 12.4	+35%	<0.01	+0%	0.9
CNR	27.5 ± 10.6	38.3 ± 14	39.5 ± 11.2	+44%	<0.01	+3%	0.62
Close to bones							
Image signal (HU)	371.3 ± 86.4	486 ± 117.1	647.8 ± 121.4	+74%	<0.01	+33%	<0.01
Image noise (HU)	11.7 ± 2.7	11.9 ± 3.3	15.8 ± 3.2	+35%	<0.01	+33%	<0.01
SNR	33.4 ± 11.3	43.1 ± 15.3	42.7 ± 12.6	+28%	<0.01	−1%	0.68
CNR	30.9 ± 11.4	40.6 ± 11.7	44.8 ± 11.7	+45%	<0.01	+10%	0.08
Intra-dural arteries							
Image signal (HU)	345.1 ± 79.1	450.1 ± 107.4	618.7 ± 116	+79%	<0.01	+37%	<0.01
Image noise (HU)	14.2 ± 4.7	13.9 ± 3.5	20.6 ± 5.5	+45%	<0.01	+48%	<0.01
SNR	26.2 ± 8.9	34.5 ± 12.7	32 ± 10.1	+22%	<0.01	−9%	0.07
CNR	27.1 ± 10.3	35.9 ± 9.0	36.0 ± 9.2	+33%	<0.01	+0%	0.95
All vascular segments							
Image signal (HU)	358 ± 90.2	456.6 ± 113.5	625.9 ± 118.5	+75%	<0.01	+37%	<0.01
Image noise (HU)	12.6 ± 3.6	12.6 ± 3.2	17.7 ± 4.9	+40%	<0.01	+40%	<0.01
SNR	30.3 ± 11.1	38.7 ± 14.7	37.9 ± 12.3	+25%	<0.01	−2%	0.4
CNR	28.9 ± 9.5	38.1 ± 13.3	39.6 ± 10.4	+37%	<0.01	+4%	0.76

AO: Aortic arch; CCA: common carotid artery; ICAc: cervical internal carotid artery; ICAp: intrapetrous internal carotid artery; ICAt: terminal internal carotid artery; MCA: middle cerebral artery; V1, V2, V3, and V4: vertebral artery, four portions; BA, basilar artery.

**Table 6:** Quantitative image analysis at regional levels of low-kVp protocol with DLR vs. higher-kVp protocol with H-IR.

Parameters	120 kVp + H-IR	100 kVp + H-IR	80 kVp + DLR	80 kVp + DLR vs.			
				120 kVp + H-IR		100 kVp + H-IR	
				% Change	*p*-Value	% Change	*p*-Value
Close to the aorta							
Image signal (HU)	382.8 ± 115.8	474.3 ± 127.7	595.8 ± 122.7	+56%	<0.01	+26%	<0.01
Image noise (HU)	18.9 ± 4.7	19.4 ± 6.1	15.6 ± 3.1	−17%	<0.01	−20%	<0.01
SNR	21.9 ± 9.9	28.1 ± 15.8	39.7 ± 12.4	+81%	<0.01	+41%	<0.01
CNR	20.5 ± 8.9	25.3 ± 8.0	39.5 ± 11.2	+92%	<0.01	+56%	<0.01
Close to bones							
Image signal (HU)	384.2 ± 95.2	490.2 ± 117.4	647.8 ± 121.4	+69%	<0.01	+32%	<0.01
Image noise (HU)	16.9 ± 4.8	17 ± 4.6	15.8 ± 3.2	−7%	0.1	−7%	0.05
SNR	24.2 ± 8.4	30.7 ± 11.2	42.7 ± 12.6	+76%	<0.01	+39%	<0.01
CNR	22 ± 7.8	27.6 ± 6.3	44.8 ± 11.7	+104%	<0.01	+62%	<0.01
Intra-dural arteries							
Image signal (HU)	366.7 ± 79.3	456.4 ± 106.9	618.7 ± 116	+69%	<0.01	+36%	<0.01
Image noise (HU)	18 ± 5.5	18.3 ± 5	20.6 ± 5.5	+14%	<0.01	+13%	<0.01
SNR	22.1 ± 8.5	26.5 ± 8.8	32 ± 10.1	+45%	<0.01	+21%	<0.01
CNR	21.7 ± 6.8	26.0 ± 6.0	36.0 ± 9.2	+65%	<0.01	+38%	<0.01
All vascular segments							
Image signal (HU)	382.3 ± 98.6	468 ± 118.5	625.9 ± 118.5	+64%	<0.01	+34%	<0.01
Image noise (HU)	17.5 ± 5.2	18.1 ± 5.4	17.7 ± 4.9	+1%	0.7	−2%	0.3
SNR	23.8 ± 9.7	28.4 ± 12.5	37.9 ± 12.3	+59%	<0.01	+33%	<0.01
CNR	22.1 ± 7.5	26.9 ± 9.5	39.6 ± 10.4	+79%	<0.01	+47%	<0.01

CCA: common carotid artery; ICAc: cervical internal carotid artery; ICAp: intrapetrous internal carotid artery; ICAt: terminal internal carotid artery; MCA: middle cerebral artery; V1, V2, V3, and V4: vertebral artery, four portions; BA, basilar artery.

**Table 7:** Qualitative image evaluation including mean scores of confidence, artifacts, sharpness, and noise with AIDR 3D (H-IR) and AiCE (DLR). Regional levels: close to the aorta (AO, CCA, V1), close to bones (ICAp, V2, and V3), and intracranial arteries (ICAt, MCA, V4, BA). CCA: common carotid artery; ICAc: cervical internal carotid artery; ICAp: intrapetrous in-ternal carotid artery; ICAt: terminal internal carotid artery; MCA: middle cerebral artery; V1, V2, V3, and V4: vertebral artery, four portions; BA, basilar artery.

Parameters	120 kVp			100 kVp			80 kVp			All kVp		
	H-IR	DLR	*p*-Value	H-IR	DLR	*p*-Value	H-IR	DLR	*p*-Value	H-IR	DLR	*p*-Value
Close to the aorta												
Confidence	4.3 ± 0.7	4.8 ± 0.4	<0.01	4.2 ± 0.6	4.7 ± 0.5	<0.01	3.6 ± 0.8	4.4 ± 0.6	<0.01	4 ± 0.8	4.6 ± 0.5	<0.01
Artifacts	3.9 ± 1.1	4.1 ± 1	<0.01	3.9 ± 1.1	4.1 ± 1	<0.01	2.5 ± 1.2	2.9 ± 1.1	<0.01	3.4 ± 1.3	3.7 ± 1.2	<0.01
Sharpness	3.6 ± 0.6	4.8 ± 0.4	<0.01	3.8 ± 0.5	4.8 ± 0.4	<0.01	3 ± 0.7	4.5 ± 0.5	<0.01	3.4 ± 0.7	4.7 ± 0.5	<0.01
Noise	3.2 ± 0.6	4.5 ± 0.5	<0.01	3.3 ± 0.6	4.8 ± 0.4	<0.01	3 ± 0.5	4.9 ± 0.3	<0.01	3.1 ± 0.6	4.7 ± 0.4	<0.01
Close to bones												
Confidence	4.5 ± 0.6	4.9 ± 0.3	<0.01	4.3 ± 0.7	4.9 ± 0.3	<0.01	4.2 ± 0.7	4.9 ± 0.3	<0.01	4.3 ± 0.7	4.9 ± 0.3	<0.01
Artifacts	4.5 ± 0.6	4.9 ± 0.4	<0.01	4.4 ± 0.7	4.9 ± 0.3	<0.01	4.3 ± 0.7	4.9 ± 0.3	<0.01	4.4 ± 0.7	4.9 ± 0.3	<0.01
Sharpness	3.6 ± 0.5	4.7 ± 0.5	<0.01	3.7 ± 0.5	4.8 ± 0.4	<0.01	3.5 ± 0.5	4.9 ± 0.3	<0.01	3.6 ± 0.5	4.8 ± 0.4	<0.01
Noise	3.4 ± 0.6	4.8 ± 0.4	<0.01	3.6 ± 0.5	4.8 ± 0.4	<0.01	3.4 ± 0.5	4.9 ± 0.3	<0.01	3.5 ± 0.5	4.8 ± 0.4	<0.01
Intra-dural arteries												
Confidence	4.6 ± 0.5	5 ± 0.2	<0.01	4.4 ± 0.6	4.9 ± 0.3	<0.01	4.3 ± 0.7	4.9 ± 0.4	<0.01	4.4 ± 0.6	4.9 ± 0.3	<0.01
Artifacts	5 ± 0.2	5 ± 0.2	-	4.9 ± 0.4	5 ± 0.2	0.3	4.8 ± 0.4	5 ± 0.2	0.04	4.9 ± 0.3	5 ± 0.2	0.02
Sharpness	3.7 ± 0.5	4.7 ± 0.5	<0.01	3.8 ± 0.4	4.8 ± 0.4	<0.01	3.4 ± 0.6	4.8 ± 0.5	<0.01	3.6 ± 0.5	4.8 ± 0.5	<0.01
Noise	3.4 ± 0.6	4.9 ± 0.4	<0.01	3.6 ± 0.5	4.9 ± 0.3	<0.01	3.4 ± 0.5	5 ± 0.2	<0.01	3.4 ± 0.5	4.9 ± 0.3	<0.01
All vascular segments												
Confidence	4.5 ± 0.6	4.9 ± 0.3	<0.01	4.4 ± 0.7	4.9 ± 0.3	<0.01	4 ± 0.6	4.9 ± 0.3	<0.01	4.3 ± 0.7	4.9 ± 0.3	<0.01
Artifacts	4.3 ± 0.8	4.6 ± 0.5	<0.01	4.1 ± 0.8	4.4 ± 0.7	0.03	3.5 ± 0.8	4.1 ± 0.8	<0.01	4 ± 0.8	4.4 ± 0.7	<0.01
Sharpness	3.7 ± 0.5	4.7 ± 0.5	<0.01	3.8 ± 0.4	4.8 ± 0.4	<0.01	3.4 ± 0.6	4.9 ± 0.3	<0.01	3.6 ± 0.5	4.8 ± 0.4	<0.01
Noise	3.4 ± 0.6	4.8 ± 0.4	<0.01	3.6 ± 0.5	4.8 ± 0.4	<0.01	3.3 ± 0.5	5 ± 0.2	<0.01	3.4 ± 0.5	4.8 ± 0.4	<0.01

## Data Availability

The data presented in this study are available from the corresponding author upon reasonable request.

## Supplementary Materials

The following supporting information can be downloaded at: https://www.mdpi.com/article/10.3390/diagnostics12051287/s1, Supplemental S1: Comparison of the two reconstruction techniques at segmental levels with constant kVp value.; Supplemental S2: Quantitative image analysis at segmental levels of low-kVp protocol with DLR vs. higher-kVp protocol with DLR.; Supplemental S3: Quantitative image analysis at segmental levels of low-kVp protocol with DLR vs. higher-kVp protocol with H-IR.
